# Learning on the IGT follows emergence of knowledge but not differential somatic activity

**DOI:** 10.3389/fpsyg.2013.00687

**Published:** 2013-10-04

**Authors:** Gordon Fernie, Richard J. Tunney

**Affiliations:** ^1^Division of Applied Medicine (Psychiatry), University of AberdeenAberdeen, UK; ^2^School of Psychology, University of NottinghamNottingham, UK

**Keywords:** Iowa Gambling Task, somatic marker hypothesis, somatic marker, implicit learning, conscious knowledge, reward learning

## Abstract

The importance of unconscious autonomic activity vs. knowledge in influencing behavior on the Iowa Gambling Task (IGT) has been the subject of debate. The task's developers, Bechara and colleagues, have claimed that behavior on the IGT is influenced by somatic activity and that this activity precedes the emergence of knowledge about the task contingencies sufficient to guide behavior. Since then others have claimed that this knowledge emerges much earlier on the task. However, it has yet to be established whether somatic activity which differentiates between advantageous and disadvantageous choices on the IGT is found before this point. This study describes an experiment to determine whether knowledge sufficient to guide behavior precedes differential autonomic activity or vice versa. This experiment used a computerized version of the IGT, knowledge probes after every 10 trials and skin conductance recording to measure somatic activity. Whereas in previous reports the majority of participants end the task with full conceptual knowledge of the IGT contingencies we found little evidence in support of this conclusion. However, full conceptual knowledge was not critical for advantageous deck selection to occur and most participants had knowledge sufficient to guide behavior after approximately 40 trials. We did not find anticipatory physiological activity sufficient to differentiate between deck types in the period prior to acquiring this knowledge. However, post-punishment physiological activity was found to be larger for the disadvantageous decks in the pre-knowledge period, but only for participants who displayed knowledge. Post-reward physiological activity distinguished between the advantageous and disadvantageous decks across the whole experiment but, again, only in participants who displayed knowledge and then only in later trials following their display of knowledge.

## Introduction

The Iowa Gambling Task (IGT, Bechara et al., 1994) was developed to model complex and uncertain choice environments in a laboratory setting. In it participants make a series of selections from four decks of cards in order to make as much, or lose as little, money as possible. Each deck pays money but all decks also contain losses. The critical aspect of the IGT is that the decks are set up so that those with the highest immediate payoffs have the highest cumulative losses such that their repeated selection will result in an overall loss. Participants must learn to avoid selecting from these decks.

Bechara et al. (1996) suggested a role for emotional processing in learning on the IGT. They reported that autonomic activity which preceded deck selections (anticipatory Skin Conductance Responses or aSCRs) differentiated between advantageous and disadvantageous decks as healthy participants learned to select advantageously on the IGT. In an influential paper Bechara et al. (1997) suggested that this differential autonomic activity preceded participants' ability to report any idea about a successful strategy to pursue on the task. Participants were defined as having a “hunch” if they could express the idea that decks A and B were riskier (or C and D were safer) but not articulate explicitly why. If they could detail why A and B were riskier (or C and D were safer) they had “conceptual” knowledge. Bechara et al. (1997) found that, on average, healthy participants entered the “hunch” period by the fourth questioning (after trial 50, although the range was between trials 30 and 80) and the “conceptual” period by the seventh questioning (following trial 80 with a range of 60–90). Bechara et al. reported that anticipatory SCRs for the disadvantageous decks were larger relative to the advantageous decks and claimed that this difference emerged in normal participants approximately between trials 10 and 50, before participants could articulate any knowledge of differences between deck types. However, although significant differences in choices from deck types developed, the difference in aSCR between deck types was never statistically significant. Patients with ventromedial prefrontal cortex damage did not show this differential aSCR activity and preferred the disadvantageous decks leading Bechara et al. (1997) to conclude that the autonomic activity was necessary to choose advantageously on the IGT and, further, as the difference in it preceded any consciously available knowledge, that the autonomic activity acted as an unconscious bias that guided behavior.

Subsequent studies have suggested autonomic activity and IGT performance are related (Bechara et al., 1999, 2000, 2002; Carter and Smith Pasqualini, 2004; Crone et al., 2004) while others have failed to find a link (Tomb et al., 2002; Campbell et al., 2004). But the interpretation of Bechara et al.'s (1997) results has not been without challenge. The main criticism rests on when participants have knowledge about the task contingencies sufficient to guide behavior. Maia and McClelland (2004) replicated Bechara et al.'s (1997) study and asked a separate group of participants more specific questions than used by Bechara et al. (1997). This group had consciously available knowledge sufficient to guide their choices much earlier than reported by Bechara et al. (1997). Crucially, this knowledge was present prior to the point at which Bechara et al. reported that differential aSCR activity emerged. This suggested that participants' behavior could be based on explicit knowledge of the likely contingencies and, therefore, did not require an explanation dependent on unconscious somatic activity. However, Maia and McClelland did not themselves record autonomic activity and so their data cannot rule out the possibility that differential autonomic activity preceded knowledge about the task contingencies.

The relative importance of knowledge about the IGT contingencies vs. autonomic activity has been examined in numerous studies. However, none have directly replicated Maia and McClelland's (2004) methods to examine the changes in participants' knowledge and autonomic activity as they complete the IGT. Gutbrod et al. (2006) measured autonomic activity and knowledge using Bechara et al.'s (1997) general questions every twenty trials in amnesic patients and healthy controls. While their controls learned to select advantageously and achieved hunch knowledge about the IGT, their patients did not. This advantageous selection occurred well before differential aSCRs emerged. Gutbrod et al. (2006) argued that their results demonstrated that knowledge about the task contingencies was the key to success on the IGT as the amnesic patients did not acquire knowledge, select advantageously or generate differential anticipatory autonomic activity but post-punishment SCRs did differentiate between deck types. However, Gutbrod et al.'s method introduced a delay between selection and feedback that may have made the task extremely difficult for amnesic patients. Without such long delays amnesic patients can learn to select advantageously on the IGT (Turnbull and Evans, 2006). Unfortunately, Gutbrod et al. (2006) did not detail when controls' knowledge emerged. But, like Maia and McClelland (2004), Evans et al. (2005) found healthy participants differentiated between deck types at above chance levels after only 20 trials.

Persaud et al. (2007) explored knowledge of deck contingencies on the IGT using post-decision wagering (PDW) as a novel measure of awareness. Their results suggest that the difference in the questions used by Bechara et al. (1997) and Maia and McClelland (2004) results in earlier awareness of the contingencies when Maia and McClelland's specific questions are used. Interestingly, in Persaud et al. (2007) the emergence of advantageous PDW closely corresponds to when Bechara et al. suggest their participants possessed conceptual, rather than hunch, knowledge of the deck contingencies when general questions are used, whereas with more specific questioning advantageous PDW is closer to when Maia and McClelland found hunch level knowledge. However, neither question style affected the time at which behavioral preference for the advantageous decks emerged nor did it appear to affect overall performance on the IGT. These results raise the possibility that IGT selection behavior does not simply follow acquisition of knowledge of deck contingencies, as suggested by Maia and McClelland's results, and so opens the possibility that autonomic activity separately influences behavior.

Guillaume et al. (2009) recorded skin conductance responses and heart rate during the IGT and explored knowledge using methods similar to Maia and McClelland's specific questions. However, knowledge was only examined at the end of the task rather than concurrently. Thus, Guillaume et al. (2009) were unable to determine when knowledge of the task contingencies emerged and if it influenced autonomic activity. They did report that participants with more accurate knowledge of the contingencies selected more advantageously than those with less accurate knowledge; that participants generated larger anticipatory SCRs before selecting from the disadvantageous vs. the advantageous decks; and IGT performance was positively correlated with the difference in this autonomic response and with degree of knowledge but the latter measures were uncorrelated.

Other researchers have examined the relationship between autonomic activity and explicit contingency knowledge using post-task questionnaires. Suzuki et al. (2003) found differential aSCR activity in the first 40 trials, replicating Bechara et al. (1996, 1997), but no differences in ratings of deck riskiness between groups split *post-hoc* on their post-selection SCR levels, implying no relationship between knowledge and SCR levels. Kleeberg et al. (2004) found aSCR and post-punishment SCRs started at a higher level and increased faster in their healthy comparison group compared to patients with MS. The healthy controls learned faster but there was no correlation with autonomic activity. Patients were generally correct when asked which decks it was best to avoid but less neurologically impaired patients made fewer disadvantageous selections and their aSCRs increased across the task leading the authors to conclude that since knowledge equated between patient groups, but somatic activity did not, cognitive appraisal was not sufficient to account for advantageous IGT behavior. But to reiterate, *post-hoc* questioning cannot inform on when awareness develops. Instead, an examination of contingency knowledge and autonomic activity is required to determine whether the two are dissociable. To this end we report an experiment using the method of assessing awareness described by Maia and McClelland (2004) along with a measure of autonomic activity derived from skin conductance recording. Our aim is to determine whether knowledge sufficient to guide behavior precedes differential autonomic activity or vice versa.

## Materials and methods

### Design

The experiment was a replication of Maia and McClelland's (2004) study with the addition that skin conductance responses were measured. A mixed-design was used with Question Group (General or Specific) a between-subjects factor, and Block of trials a within-subjects factor. Three dependent measures were obtained: participants' deck selections on the IGT, participants' knowledge of the task contingencies, and the change in participants' physiological arousal prior to card selection (aSCRs) and following card selection (r or pSCRs; reward or punishment SCRs).

### Procedure

On arrival for testing participants were given a brief description of the task, an account of what was involved in the recording of electrodermal activity, and in the General Question Group, information about the recording of their answers using a tape recorder. These participants were told that questions would appear on the computer screen periodically throughout the task and they must speak their answers into the tape recorder. It was emphasized to all participants that the experimenter would not interact with them nor answer any questions about the task after the opportunity to ask questions about instructions had ended (following their acknowledgement that they understood the task instructions). Informed consent was obtained from all participants.

The index and middle fingers of participants' left hands were cleaned using an alcohol free wet-wipe. Once dry an isotonic (0.5% saline) gel (Biopac Gel 101) was rubbed into the skin of the medial phalanges of the index and middle fingers of participants' left hand before the MP30 electrodes were attached. Participants were instructed that it was important to stay as still as possible throughout the experiment and to make themselves comfortable so that they only moved their right hand when controlling the mouse, and in the Specific Question Group, when they entered answers using the keyboard.

Participants then read the task instructions. These were exactly the same as those used in the Bechara et al. (1999, 2000; Fernie and Tunney, 2006) with the addition of information about the periodic interruptions in which questions would be asked. A period of at least 5 minutes was allowed to elapse from electrode attachment to task commencement to allow the electrode gel time to be absorbed into each participant's skin. During this time participants were informed that the experimenter would be present in the room but would not be monitoring their performance. Participants were told that the purpose of the experimenter's presence was to monitor the SCR record and, in the General Question Group, to operate the tape recorder when required. They were told that there would be no interaction with the experimenter except if, in the Specific Question Group, clarification was needed on the terms used in the questionnaire. Participants were then reminded that the most important thing was to earn as much money as possible, or to avoid losing as much as possible.

SCRs were recorded without interference until the task ended. The experiment began once a visual inspection indicated that the apparatus was reliably recording electrodermal activity. An on-screen message instructing the participants to consider which deck they would like to choose. No decks could be selected while this message was on-screen. After 5 seconds another message appeared telling participants to “Please select a card.” The mouse pointer re-appeared and the decks became active. The 5 seconds prior to deck choice constituted the period during which SCRs were considered to be anticipatory. Following the selection of a card the computer displayed the amount won accompanied by the sound of a man shouting “Yippee!” This sound was marked on an analog channel of the SCR record and allowed the accurate pinpointing of SCR events in relation to deck choices. One second after the reward, the amount lost was displayed accompanied by the sound of a man shouting “Doh!” The reward and loss information remained on-screen for 5 seconds. The instruction to “Consider your next choice” was then displayed for 5 seconds before participants were again instructed to choose a card. SCRs in the 5 seconds following deck selection were considered to be post-selection SCRs. Therefore, the inter-trial interval was at least 12 seconds but varied depending on how long participants took to choose their next card following the instruction to do so.

The experiment concluded following 100 trials on the IGT and when participants' task knowledge had been probed nine times. The length of time that the experiment took differed between participants and was dependent on the speed with which they selected cards and answered the questions. As there were more questions in the specific question group these participants tended to take longer. The experiment took around 1 h and although participants were told the prospective length of the task this information could provide no hint about when it would end.

On completion of the task all electrodes were removed and participants were fully debriefed. Each participant received the amount they had earned on the task plus an additional £2.

### Participants

Thirty-two predominantly post-graduate students were recruited from the University of Nottingham community via posters, online advertisements, and direct email to members of a participant pool. The volunteers were told that they would be participating in a cognitive task and have the opportunity to earn up to £12. They were told that some physiological measures would be recorded and that the experiment took approximately 1 h. Sixteen participants were randomly assigned to each question group [the General questions of Bechara et al. (1997); or the Specific questions of Maia and McClelland (2004)]. The mean age was 25.68 (σ_M_ = 1.22) in the Specific question group and 24.63 (σ_M_ = 0.92) in the General question group. There were nine male participants in the Specific and seven in the General question group.

### Apparatus—behavioral task

A computerized version of the IGT with the hint instructions and real money incentives was used (Fernie and Tunney, 2006). Breaks in the behavioral task occurred after the first twenty trials and from then on after each ten trial block so that participants' knowledge could be probed using the condition-specific questions. More detail on these is provided below. The addition of questionnaires and skin conductance recording resulted in the task taking around 1 h to complete. As this experiment took on average four times longer than the previous purely behavior studies used in Fernie and Tunney (2006), the value of the payoffs was increased to four times the amount. Therefore, wins increased from 10p to 40p in decks A and B, and from 5p to 20p in decks C and D. All values for losses increased similarly.

### Apparatus—knowledge probes

The administration and structure of the questionnaires followed the procedure of Maia and McClelland (2004). Briefly, the task was interrupted after twenty trials and thereafter after every ten trials when instructions on the computer screen informed participants that they would be asked some questions about the task. In the Specific Question group participants were given the detailed questionnaire as used in Maia and McClelland (2004). The questionnaire was computer-based and required selection of options using the mouse or entry of answers using the numerical keypad. Three measures of knowledge were obtained for each deck at each question period: a deck rating from −10 to 10 (Deck Rating), an estimate of the average net amount won or lost on the deck (Estimated Net) and a calculated net amount based on participants' estimates of how much they would win, how often they lost, and how much that average loss was (Calculated Net). The participants were also asked which deck they would choose if they only had one choice (One Deck).

In the General Question group participants were presented with the two questions used by Bechara et al. (1997) on subsequent screens: “Tell me all that you know about what is going on in this game” and “Tell me how you feel about this game.” Participants' responses were recorded using a tape recorder operated by the experimenter who sat behind a large dividing screen in the same room as the participant. The questions were presented on-screen to minimize any potential experimenter influence and to equate the two question conditions. Interaction with the experimenter was kept to a minimum and was initially restricted to prompting participants to answer the question before them. However, some participants' answers were so minimal that some additional prompting was occasionally required. In the main this took the form of directing participants' answers to their knowledge of the decks.

The presentation and cessation of the questions in both conditions was accompanied by a computer beep to mark the beginning and end of the question period on the skin conductance record, and to inform the experimenter when to start and end the tape recorder in the General question condition.

### Apparatus—analysis of general group transcripts

Verbal responses to questions were transcribed from the tape recording. Three post-graduate students, naïve to the experimental hypothesis, were recruited and paid to assess the transcripts and classify the knowledge displayed at each question period using Maia and McClelland's (2004) decision tree. The assessors first undertook training on the decision tree using sample answers created to cover all possible outcomes from the tree. One hundred percent accuracy was required before the actual transcripts were assigned. When the sample transcripts were not correctly rated the assessor was told and asked to try again. Most raters accurately rated each transcript on their first attempt. Rarely were three attempts required, but following correct answers the assessor had to convince the experimenter (GF) of why they had reached the assessment they had.

Once the actual transcripts had been assessed the assessors met to compare results. Any disagreements on any of the participant's answers were debated until a unanimous decision among the assessors was reached. If this was not possible a majority decision for that answer was used. These assessments of participants' answers were used to determine when knowledge was displayed in the General Question group.

### Classification of knowledge

Maia and McClelland's (2004) attempt to replicate Bechara et al.'s (1997) study was hampered by the lack of detail about how Bechara et al. assessed knowledge and categorized it into two (hunch and conceptual) of their four knowledge periods. Maia and McClelland (2004) developed a detailed solution to resolve this that resulted in a decision tree to categories each participants' knowledge at each question period into one of the six knowledge categories possible on the IGT. These are: no professed knowledge, incorrect or incomplete hunch/knowledge, partial hunch, hunch, partial conceptual, and conceptual. Even with this decision tree there were still several ways knowledge could be assessed in order to integrate it into Bechara et al's knowledge periods. This integration is effectively along two axes. The first concerns whether knowledge expressed about only one of the good decks is included as conceptual knowledge (partial conceptual). In a strict interpretation of Bechara et al's criteria partial conceptual knowledge would not count as conceptual knowledge because it is not full understanding of both good decks—Maia and McClelland (2004) called this grouping “both.” In the “partial” grouping partial conceptual knowledge is included in the conceptual period.

The second axis in integration of the two knowledge assessment systems concerns when participants first show any level of knowledge. A conservative approach would only count knowledge expressed consistently throughout all question periods from the one where it was first expressed through each subsequent questioning i.e., if upon reaching one level of knowledge the participant never returned to a lower state of knowledge. An aggressive interpretation would allow an earlier expression of knowledge to be counted even if later questioning revealed that this level of knowledge was no longer being expressed at a later question period. Maia and McClelland's aggressive, “partial” grouping best fit Bechara et al.'s (1997) results. However, Maia and McClelland focused on the “both” grouping as it more reflected Bechara et al.'s (1997) classification of conceptual knowledge.

These terms are detailed here as they will provide different answers for the question of when knowledge emerges with an aggressive approach using the partial grouping likely providing an earlier point than a conservative approach using the both grouping. Each participant's knowledge at each measurement was independently assessed but mean results within groups will be compared with the results of Bechara et al.'s (1997) and Maia and McClelland (2004) criteria and the closest matching group averages used.

### Apparatus—electrodermal activity

A BIOPAC Systems MP30 system running on a Macintosh computer was used to record electrodermal activity. Skin conductance was recorded at 10 Hz using two Ag/AgCl electrodes connected to the volar surfaces of the medial phalanges on participants' index and middle fingers of the left hand (all participants were right handed). Because the MP30 system does not have the facility for a direct link between the recording computer and the task presentation computer, marking the occurrence of events was achieved by recording the sounds produced on the task presentation computer during the task. These sounds were recorded by the MP30 via an analog input. During the IGT gains and losses were accompanied by concurrent auditory stimuli. These served as markers for events in this experiment. Additionally, the experimenter marked the skin conductance record when an event occurred. As this measure is less reliable and not as temporally accurate it was only referred to if any ambiguity about when an event occurred existed in the auditory record.

### Skin conductance activity analysis

Skin conductance responses were analyzed using the Student Lab Pro software for the MP30 system. The first step in the analysis was the removal of the downward drift in the SCR record. A mathematical transformation provided by the Student Lab software was used to remove it prior to analysis. This “difference” transformation measures the difference in amplitude between two data samples separated by a particular number of points (in this case it was 10). The difference is then divided by the time interval between the two samples.

The SCRs were analyzed using the area-under-the-curve measurement. This measurement calculates the total area between a waveform and a baseline value within the endpoints of a selected area. In effect a line is drawn between the user defined start and end points of the waveform. For anticipatory SCRs this was the 5 seconds prior to deck choice as determined by the auditory signal's mark on the analog channel. For post-selection SCRs the start point was 1 second after this marker and the end point was again 5 seconds later. These area-under-the-curve measurements were then divided by the time interval to give a value in amplitude units per second (μS/s).

## Results

### Behavioral data

The principle behavioral measure of interest is Mean net score which was calculated by subtracting the number of cards selected in each ten trial block from the advantageous decks A and B from the number selected from disadvantageous decks C and D. Positive scores indicate a preference for advantageous decks and an increase in the mean net score across blocks indicates that participants learn to choose from the advantageous decks during the course of the experiment.

Mean net score for the General Question group was 20.44 (*SD* = 22.06). A one sample *t*-test found that this was significantly greater than zero, *t*_(15)_ = 3.93, *SD* = 22.06, *p* < 0.01 indicating that participants in this condition showed an overall preference for the advantageous decks. The same was true of participants in the Specific Question group. Their mean net score was 28.56 (*SD* = 29.04) and this was significantly greater than zero, *t*_(15)_ = 3.71, *SD* = 29.04, *p* < 0.01.

**Figure 1:**
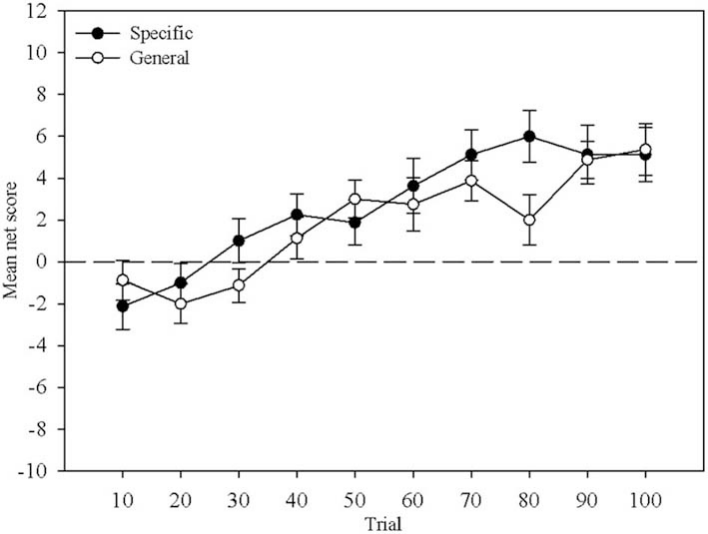
**Mean net score across 10-trial blocks.** The closed circles represent the Specific question group and the open circles represent the General question group. Error bars are the standard error of the mean.

Mean net score was calculated for each block of ten trials and compared between Question Group and across Block. Figure 1 displays this comparison. A mixed-design ANOVA revealed no main effect of Question Group, *F*_(1, 29)_ < 1. There was a main effect of Block, *F*_(4.31, 124.89)_ = 15.43, *MSE* = 44.26, *p* < 0.01, that reflects the increase in mean net score with more trials, but no interaction, *F*_(4.31, 124.89)_ = 1.53, *MSE* = 29.0, *p* > 0.05 indicating that learning proceeded at a similar pace in both question groups. This replicates Maia and McClelland's (2004) result demonstrating that the nature of the questions participants received did not differentially affect their behavior.

### Knowledge of the task: general question group

The independent ratings suggested at least half the participants reached what Bechara et al. described as the Conceptual Period but this depended on the method of classifying conceptual knowledge (Table 1). Like Maia and McClelland (2004) the aggressive approach provided the best fit to Bechara et al.'s (1997) data and the discussion that follows will refer to this approach only. However, unlike Maia and McClelland, the “partial” rather than “both” grouping of conceptual knowledge best matched Bechara et al's data. In classifying knowledge aggressively all but one participant displayed Hunch (or in Maia and McClelland's terms level-1) knowledge and this occurred on average after 43 trials (Bechara et al.—all participants by trial 50; Maia and McClelland—88% of participants by trial 43).

**Table 1:** **Summary of participants' knowledge expression in Bechara et al. (1997); Maia and McClelland's (2004) replication condition and the General question condition of this study**.

	**Bechara et al. (1997)**	**Maia and McClelland (2004) Replication condition**		**This study General questions**	
		****		****	
		**Conservative**	**Aggressive**	**Conservative**	**Aggressive**
% participants who did not reach the hunch period:	0	37.5	12	50	6.25
Average trial in which participants had hunch knowledge:	50 (30–60)	62 (5.8)	43 (4.6)	73 (6.8)	43 (3.9)
		**Partial grouping**		**Partial grouping**	
% participants who did not reach the conceptual period:	30	47	25	62.5	31.25
Average trial in which participants had conceptual knowledge:	80 (60–90)	74 (1.6)	62 (6.6)	83 (9.2)	53 (6.2)
		**Both grouping**		**Both grouping**	
% participants who did not reach conceptual knowledge:		77	60	87.5	50
Average trial in which participants had conceptual knowledge:		91 (5.6)	72 (4.8)	83 (9.2)	55 (8.0)

Figures in parentheses are the range of observations for Bechara et al. (1997) and the standard error of the mean otherwise. The aggressive/conservative axis determines when knowledge exists. The conservative approach requires consistent knowledge expression from the first measurement through subsequent measurements. The aggressive approach does not. The both grouping requires knowledge expression that both good decks are good. The partial grouping only requires that one good deck is identified.

Classification of conceptual knowledge using the “partial” grouping fit Bechara et al.'s data better than using the “both” grouping. In this case only around 30% of participants (vs. 62.5% using the conservative approach) failed to exhibit conceptual (or level-2) knowledge. Bechara et al.'s figure was also 30% and there conceptual knowledge was achieved on average by trial 80. Using either grouping method and an aggressive approach, conceptual knowledge was achieved substantially earlier on average in our study (by 53 or 55 trials for the “partial” and “both” groupings, respectively). Maia and McClelland (2004) also found that the “partial” grouping resulted in the majority of participants (~75%) being classified as having conceptual knowledge and on average this occurred by trial 62. However, they used the “both” grouping when comparing their results to Bechara et al.'s. With the current data, the “both” grouping would decrease the proportion of participants with conceptual knowledge to 50%.

### Knowledge of the task: specific question group

**Figure 2:**
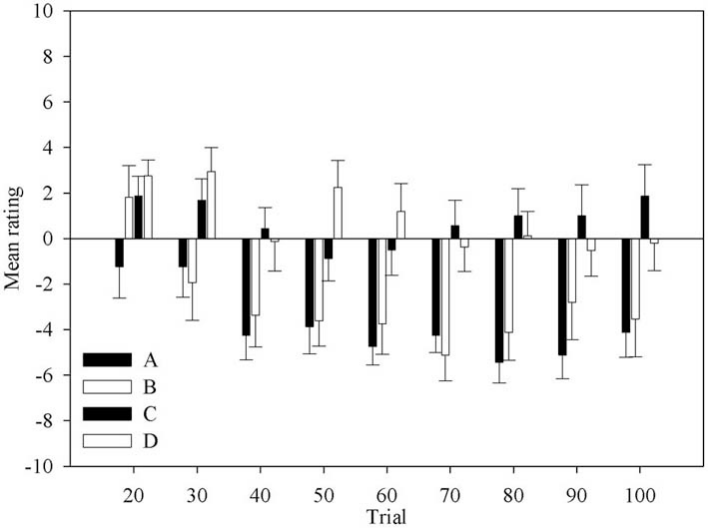
**Mean rating for each deck across question period.** Error bars are the standard error of the mean.

Figure 2 shows the change in ratings for each deck across block. The ratings are mostly negative for all decks. It is clear that most participants do not believe any of decks are good. However, it is equally clear that decks C and D are accurately perceived as being better than decks A and B. Although this indicates that participants have not fully understood the patterns of gains and losses of the decks, and thus of the task, such knowledge would be sufficient to guide behavior advantageously. This knowledge is present in most participants at the second question period. Participants also correctly rated deck A as one of the disadvantageous decks from the first opportunity they are given.

**Figure 3:**
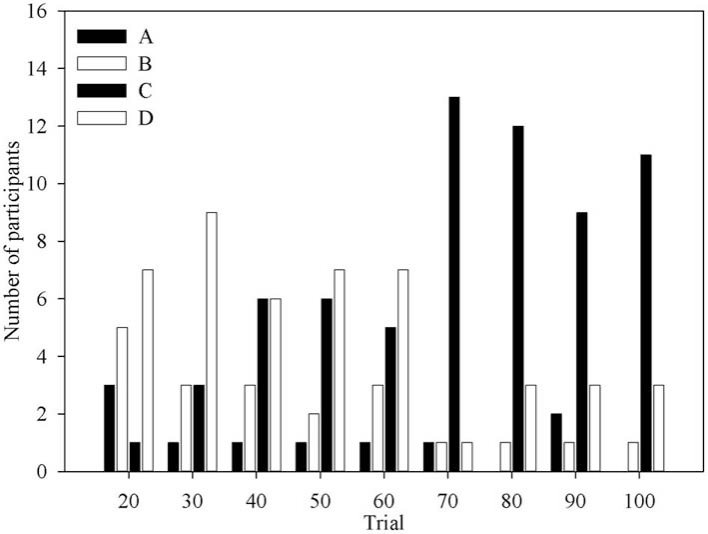
**The number of participants at each question period who selected each deck as the One Deck they would choose if forced to only pick from one**.

Figure 3 shows the number of times each deck was identified as the one deck participants would choose if they could only choose one for the remainder of the task. Aside from the first question period, when deck B is often advantageous, most participants would choose deck C or deck D. Indeed the number of participants who would choose deck C increases with experience of the task, mirroring the behavioral data in previous results (Fernie and Tunney, 2006).

**Figure 4:**
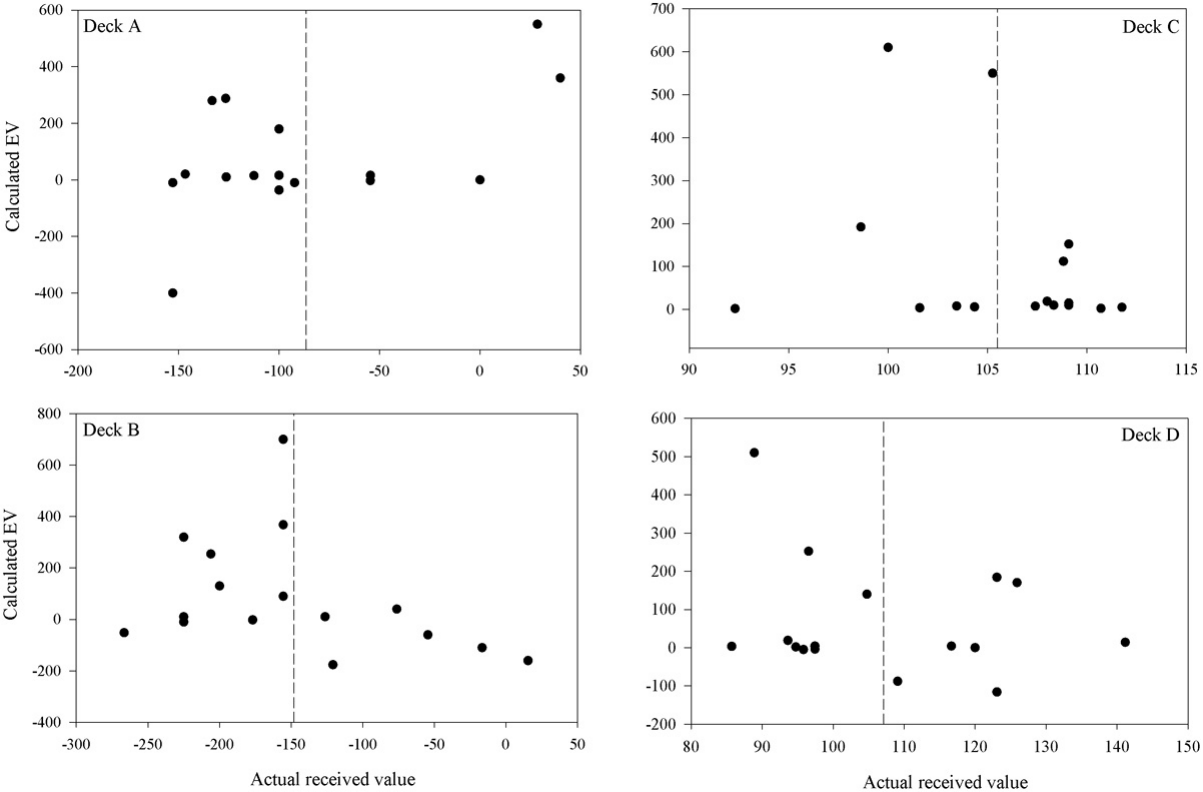
**Calculated vs. actual expected value on each deck after 100 trials for each participant (except for participant 3 for whom figures are following 80 trials).** The calculated expected value was calculated from a participant's estimates of the average gain, average loss and frequency of loss over ten selections from that deck. The dashed lines are the mean actual expected values.

**Figure 5:**
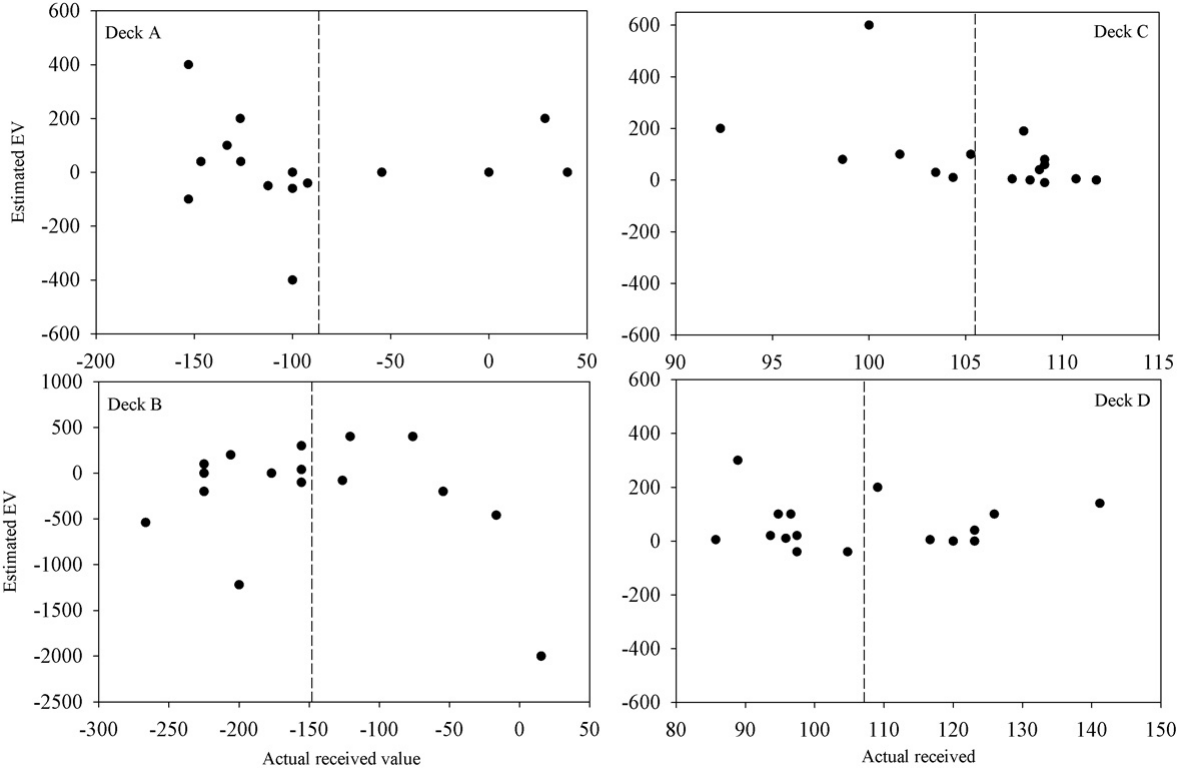
**Estimated vs. actual expected value on each deck after 100 trials for each participant (except for participant 3 for whom figures are following 80 trials).** The dashed lines are the mean actual expected values.

Participants' quantitative knowledge of the task as assessed using the Expected Net and Calculated Net measures was not good. The Estimated Net was an estimate of the average amount won or lost on the deck while the Calculated Net was calculated from participants' estimates of how much they would win, how often they lost, and how much that average loss was when selecting from each deck. Figure 4 displays the Calculated Net measure for each deck from every participant in the final question period. The dashed line shows that the mean received value for each deck is close to its pre-test expected value (decks A and B are negative; decks C and D are positive). Pearson correlations were calculated between the actual received values and each participant's Calculated Net measure from the final question period. Calculated Net measures do not correlate with the values actually received for deck B, C, or D (*r* = 0.46, 0.43, and 0.34, respectively, *p*'s > 0.05), except on deck A (*r* = 0.92, *p* < 0.01). Actual received values do not correlate with the Expected Net measure on any deck (*r* = −0.20, 0.13, 0.19, 0.05 for decks A, B, C, and D, respectively) as illustrated in Figure 5. Together these results suggest that most participants' quantitative knowledge of the deck contingencies is not accurate. Indeed for many participants the Expected or Calculated Nets are positive for decks A and B, and negative for decks C and D. This may indicate that participants are unable to retain quantitative knowledge about the decks or that they did not comprehend what was required in the answer for the measures themselves.

Table 2 displays a breakdown of when and what proportion of participants displayed knowledge of the task contingencies when actual received values are used. The One Deck and Deck Ratings questions were used to assess hunch, or level-1 knowledge, while the Expected and Calculated Net questions were used to assess conceptual or level-2 knowledge. As conceptual knowledge of the task was so poor (Figures 4, 5) and the focus of this paper is when knowledge sufficient to guide behavior emerges only the breakdown of Deck Ratings and One Deck measures will be discussed here.

**Table 2:** **Knowledge assessment for Specific question group using “partial” grouping (either deck with the highest net value at the time of questioning received the best score on each measure) or “both” grouping (both decks with the highest net value at the time of questioning received the best scores on each measure)**.

**Question type**	**“Partial” grouping**		**“Both” grouping**	
	**Conservative**	**Aggressive**	**Conservative**	**Aggressive**
**RATINGS**				
% participants who did not reach Hunch level:	20	0	50	12.5
average trial number in which they did so:	39 (6.8)	22 (1.0)	59 (7.6)	33 (3.04)
**ONE DECK**				
% participants who did not reach Hunch level:	6.25	0	as “partial” grouping as there is only one response possible	
average trial number in which they did so:	47 (7.5)	21 (0.6)		
**EXPECTED NET**				
% participants who did not reach Conceptual level:	25	0	62.5	12.5
average trial number in which they did so:	51 (7.9)	26 (2.2)	57 (9.5)	36 (4.2)
**CALCULATED NET**				
% participants who did not reach Conceptual level:	50	0	68.75	12.5
average trial number in which they did so:	65 (8.5)	26 (2.4)	72 (12.4)	36.4 (4.3)

Average trial values are rounded to the nearest trial. Values in parentheses are the standard error of the mean. When actual received values are used with a partial grouping and aggressive approach all participants reach the hunch level by around trial 20 and conceptual level by trial 26.

An aggressive approach using a “partial” grouping was used in the General Question group. This strategy suggests that all participants have hunch level knowledge by trial 22 using the Deck Ratings or by trial 21 using One Deck. More similar results to the General group are obtained by using a conservative approach and a “partial” grouping: 80% of participants have hunch level knowledge by trial 39 using the Deck Ratings or by trial 47 (93.75% of participants) using One Deck. In the analyses that follow where differences pre- and post-knowledge are considered we will use this latter strategy and the figures obtained from the Deck Ratings measure because Deck Ratings required more information from participants. Although, the strategies used to determine when knowledge was present are different in each group, we believe this is appropriate because participants showed no differences in behavior and so it can be assumed that their experience of the task was similar. We can further assume that their pre-task knowledge was similar and as their behavior did not differ, their knowledge remained similar throughout the task (though see Persaud et al., 2007). All that differed between the groups then was the specificity of knowledge probe. If this is the case then an aggressive approach is appropriate for the General group because their knowledge was not probed as effectively as the Specific group participants. Ideally, a conservative partial approach would have been used throughout but this would not have been sensitive enough in the General condition to indicate when knowledge sufficient to guide behavior appeared. The use of these two approaches results in figures for knowledge emergence that is consistent between groups and with the previous literature using the General questions. It is also consistent with the behavior shown in Figure 1. Mean net score first moves above chance in both groups in block 4, the block during which the above measures suggest participants can determine C and D to be the best decks.

**Figure 6:**
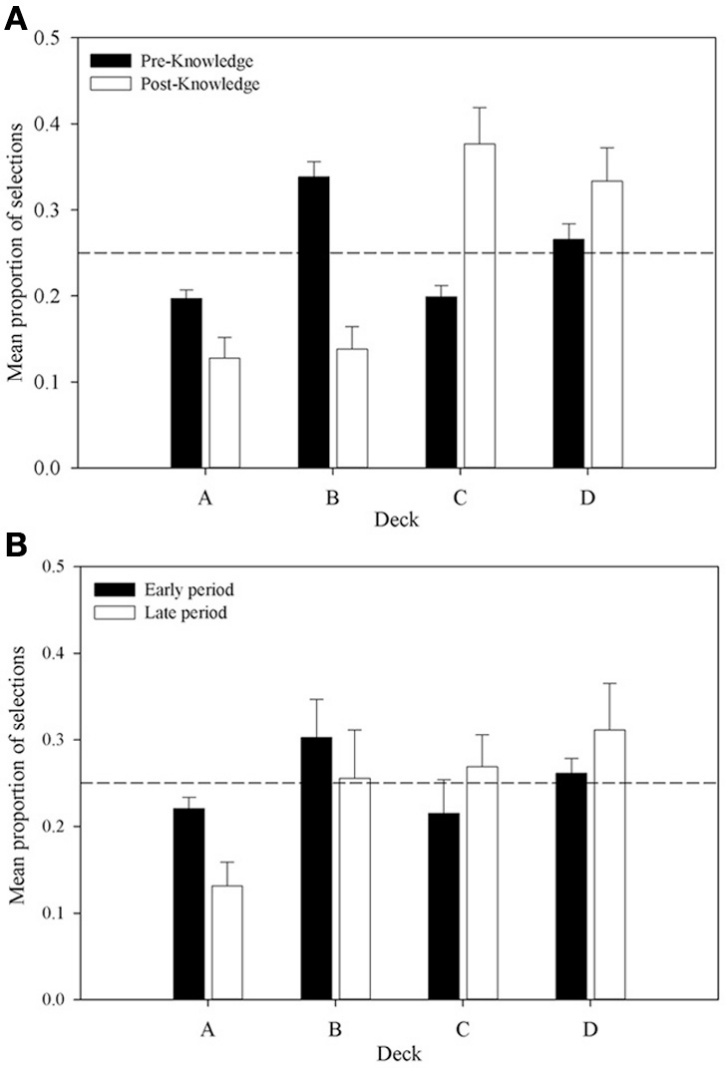
**Mean proportion of cards selected from each deck in (A) the pre- and post-knowledge periods for participants who displayed knowledge (*n* = 27), and (B) the comparable periods for participants who did not display knowledge (*n* = 5).** Error bars are the standard error of the mean. The dashed line represents chance selection.

Further support is provided by an analysis of the proportion of selections from each deck in the pre- and post-knowledge periods across all participants who were categorized as having displayed knowledge (displayed in Figure 6A). The proportion of selections from decks A and B declines from the pre- to post-knowledge period, whereas the proportion increases for decks C and D. This supports the supposition that participants' choices are guided by knowledge of the decks. A 4 × 2 (Deck by Time) repeated measures ANOVA examined these data. A significant interaction between Deck and Time was revealed, *F*_(2.28, 59.35)_ = 17.41, *MSE* = 0.03, *p* < 0.01; as was a main effect of Deck, *F*_(3, 78)_ = 7.48, *MSE* = 0.03, *p* < 0.01. There was no effect of Time, *F*_(1, 26)_ < 1. A complex interaction comparison examined the interaction between Deck Type and Time by collapsing data across advantageous and disadvantageous decks in each knowledge period. This 2 × 2 repeated measures ANOVA found a significant interaction between Deck Type and Time, *F*_(1, 26)_ = 35.60, *MSE* = 0.03, *p* < 0.001; a main effect of Deck Type, *F*_(1, 26)_ = 15.38, *MSE* = 0.03, *p* < 0.001; but no main effect of Time, *F*_(1, 26)_ = 2.09, *MSE* < 0.01, *p* > 0.05. Subsequent simple comparisons found that the proportion of advantageous choices in the pre-knowledge period was not significantly greater than the number of disadvantageous choices, *F*_(1, 26)_ = 2.41, *MSE* = 0.03, *p* > 0.05; whereas it was in the post-knowledge period, *F*_(1, 26)_ = 31.84, *MSE* < 0.01, *p* < 0.001. Figure 6A shows that, consistent with previous experiments, this difference appears to be due to changes in selections from decks B and C. In the post-knowledge period the proportion of selections from deck B has decreased below chance and the proportion of selections from deck C has increased above chance. Similar patterns are found in decks A and D, but the major changes lie in decks B and C.

A similar pattern is shown in Figure 6B for the participants who displayed no knowledge. The early period shown in the Figure represents the proportion of choices from each deck up until the mean trial at which participants in the knowledge group displayed knowledge. The late period is the period from this mean trial until the end of the task. While behavior in this group looks similar to the knowledge group, there are several differences. The proportion of selections from each deck is much closer to chance in both time periods. In the late period, unlike the participants with knowledge, selections from B are not below chance nor are selections from deck C above chance. These observations were tested in a 4 × 2 (Deck by Time) repeated measures ANOVA. It found no interaction, *F*_(1, 26)_ = 2.44, *MSE* = 0.01, *p* > 0.05; no main effect of Deck, *F*_(1, 26)_ = 1.29, *MSE* < 0.01, *p* > 0.05; and no main effect of Time, *F*_(1, 26)_ < 1. These results suggest that only with knowledge sufficient to guide behavior do participants select advantageously on the IGT replicating Maia and McClelland (2004) but contradicting Bechara et al. (1997). The next section will examine whether differences in physiological responses exist prior to knowledge being displayed and so leave an opportunity for an explanation of IGT behavior incorporating somatic markers.

### Physiological measures—aSCR

**Figure 7:**
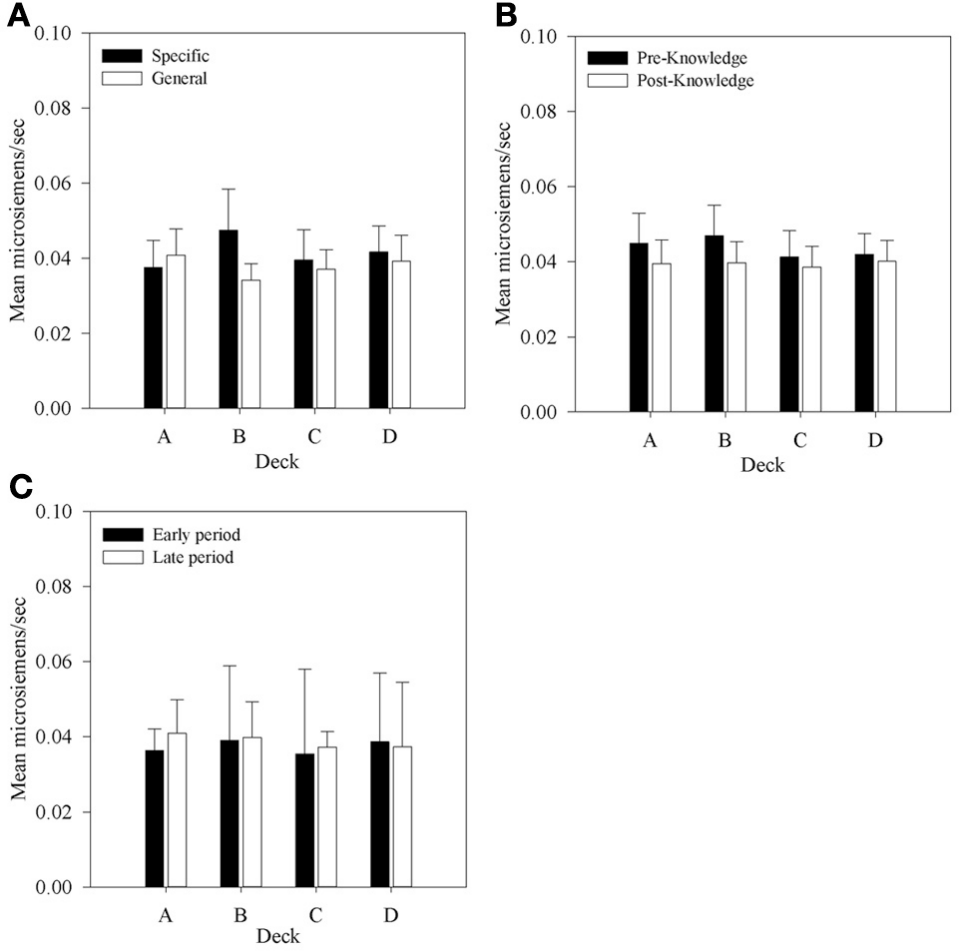
**Mean aSCRs for each deck in each group, (A) across all selections; (B) in selections prior to and following knowledge expression in those participants who displayed knowledge, and (C) the equivalent figure to b for participants who did not demonstrate knowledge—aSCRs before and after the mean trial at which knowledge was expressed in those who expressed knowledge (trial 39 in the Specific Group and trial 43 in the General group).** Error bars are the standard error of the mean.

Anticipatory SCRs were the mean area under the curve of the SCR in the 5 seconds prior to selecting a card. Mean aSCRs for each deck were obtained by taking the average aSCR for that deck for each participant and dividing across participants. These mean aSCRs are displayed by Group in Figure 7A. Figure 7A shows that mean aSCRs are generally very low and that they are similar in each Group. To determine if any differences existed, a 2 × 4 (Group by Deck) mixed-factor ANOVA was run. Although mean aSCR was higher in the Specific Question Group than in the General Question Group no main effect of Group was found, *F*_(1, 30)_ < 1. There was also no main effect of Deck, *F*_(1, 30)_ < 1. Despite the higher mean aSCR for deck B in the Specific Question Group, there was no interaction between Question Group and Deck, *F*_(3, 90)_ = 2.02, *MSE* < 0.01, *p* = 0.12. As in the behavioral analysis no differences in aSCR were found between groups nor were any differences observed between decks. This first result supports the conclusion that the different questioning did not differentially affect participants, whereas the second contrasts with the data reported by Bechara et al. (1997).

In the previous section it was determined that most participants in each group display at least hunch level knowledge of the task between trials 40 and 50. In order to determine whether aSCR differences existed between decks prior to this period, average aSCRs before and after each participant's expression of knowledge were calculated for each deck for those participants who displayed knowledge (80% in the Specific group, 93.75% in the General group). As there were no differences in aSCR between groups in the previous analysis this factor was not included in the subsequent analyses. Some participants did not select cards from some of the decks in the period following their expression of knowledge. As a result there were no SCRs on some decks for seven participants who either chose only one deck in the period after they displayed knowledge (deck C in one participant in the Specific question group), or no longer chose from both deck A or B (two participants in both groups) or did not select from deck B (two participants in the Specific question group and one in the General question group). In the analyses that follow missing values were imputed using the automatic multiple imputation method in SPSS 20.0 and the results pooled across five imputations. The resulting 4 × 2 (Deck by Time) repeated measures ANOVA found no significant effects: Deck by Time, *F*_(1.54, 40.08)_ = 2.0, *MSE* < 0.01, *p* > 0.05; Deck, *F*_(1.74, 45.13)_ = 1.50, *MSE* < 0.01; Time, *F*_(1, 26)_ < 1. The same outcome was found when participants with missing data were excluded.

As automatic SCR recording was employed it is possible that interference from SCRs following rewards or punishments affected subsequent aSCRs. If so, then larger aSCRs would be expected following a loss than following a gain. But an examination of aSCRs in each deck following a gain and a loss revealed no such difference. These data were calculated for each participant and entered into a 4 × 2 (Deck by Reinforcer Type) repeated measures ANOVA. No main effect of Reinforcer Type was found, *F*_(1, 27)_ < 1; nor was there a main effect of Deck, *F*_(1.98, 53.33)_ < 1; nor an interaction, *F*_(1.74, 46.88)^1^_ < 1. This suggests that automatic gathering of SCRs did not impact on the clarity of the physiological record.

The main purpose of this experiment was to determine if any physiological responses distinguish between decks prior to participants' expression of knowledge; that is, SCR changes in the pre-hunch period of Bechara et al. (1997). No significant differences in aSCR were found between decks before participants had knowledge of the task contingencies. This does replicate Bechara et al.'s result, and like their data the mean values found in the present study within this period, displayed in Figure 7B, suggested that a difference between decks A and B and decks C and D may exist although there was no significant interaction. Therefore, no evidence was found to support the hypothesis that differences in aSCRs precede knowledge expression in participants who express hunch level knowledge. Figure 7C shows that in participants who did not display any knowledge mean aSCRs across the same time periods were at a similar level.

### Physiological measures—post-selection SCRs

**Figure 8:**
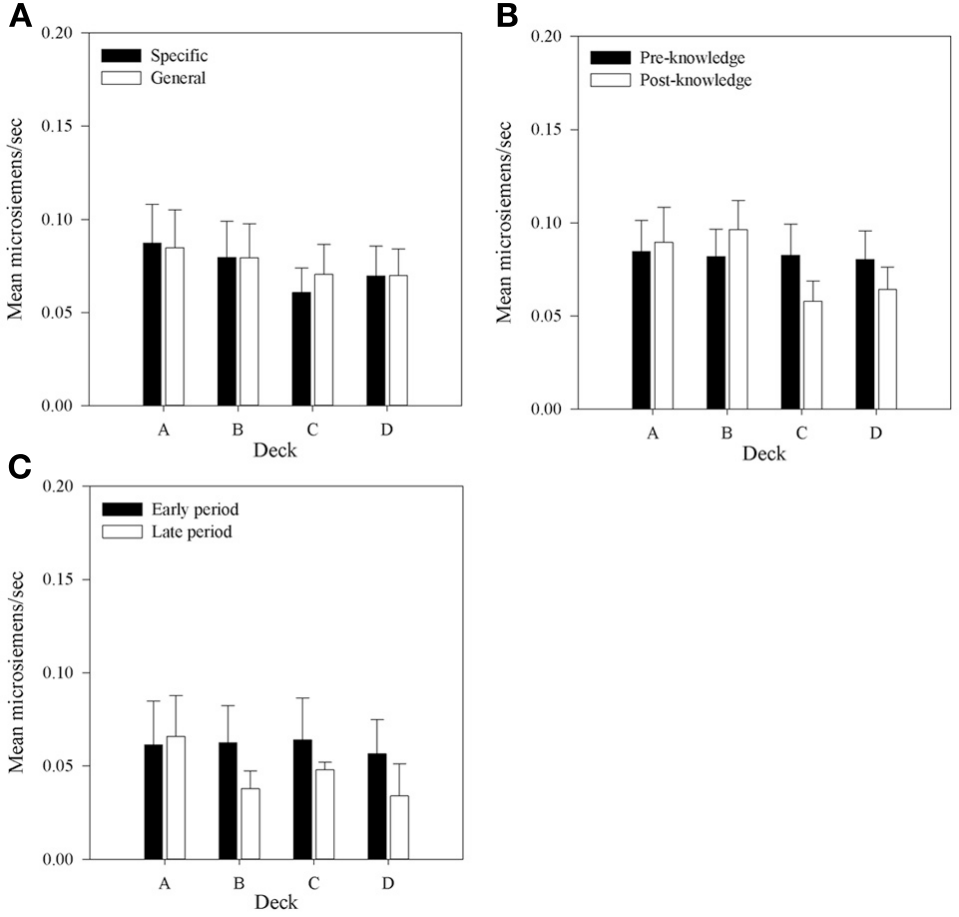
**Mean rSCRs for each deck in each group, (A) across all selections; (B) in selections prior to and following knowledge expression in those participants who displayed knowledge; and (C) the equivalent figure to b for participants who did not demonstrate knowledge—rSCRs before and after the mean trial at which knowledge was expressed in those who expressed knowledge (trial 39 in the Specific Group and trial 43 in the General group).** Error bars are the standard error of the mean.

**Figure 9:**
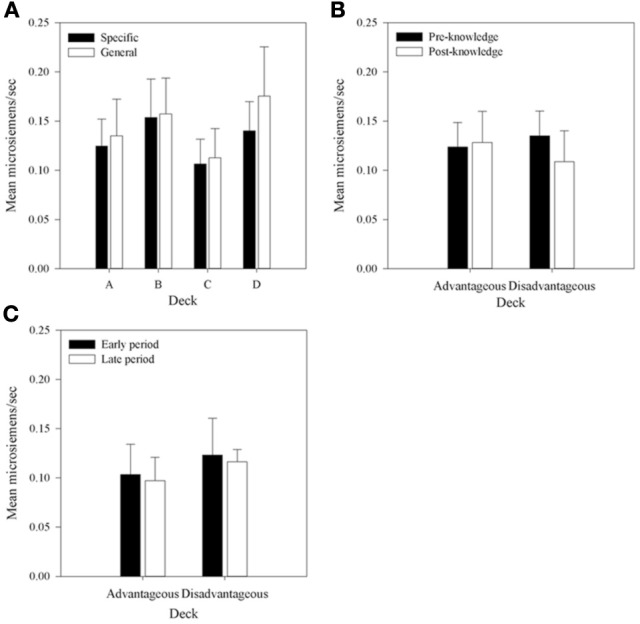
**Mean pSCRs for each deck in each group. (A)** Across all selections. **(B)** Mean pSCRs for the advantageous and disadvantageous decks in selections pre- and post-knowledge expression in those participants who displayed knowledge. **(C)** The equivalent graph to b for the participants who did not demonstrate knowledge (*n* = 5)—pSCRs in the period before and after the mean trial at which knowledge was expressed in the majority of each group. Error bars are the standard error of the mean.

Post-selection SCRs were the mean area under the curve of the SCR in the 5 seconds after a card was selected. These SCRs were split into those following a reward with no punishment (reward SCRs or rSCRs) and those following trials on which punishment occurred (punishment SCRs or pSCRs). Mean rSCR and pSCRs for each deck were calculated for each individual. The mean of these values provided the mean post-selection SCRs displayed by Group in Figures 8A, 9A for reward and punishment SCRs, respectively.

Figure 8A shows that mean rSCRs are similar in each Group but that there is a trend for rSCRs to be higher in decks A and B. A 2 × 4 (Group by Deck) mixed-factor ANOVA was run to examine rSCRs across all selections. There was no interaction, *F*_(1, 30)_ < 1; no main effect of Group, *F*_(1, 30)_ < 1; but a main effect of Deck was found, *F*_(1, 30)_ = 5.97, *MSE* < 0.01, *p* < 0.01. A planned complex main comparison was performed to investigate whether rSCRs differentiated between the advantageous and disadvantageous decks. It found that rSCRs were higher for the disadvantageous decks, *F*_(1, 30)_ = 10.12, *MSE* < 0.01, *p* < 0.01. These results are consistent with previous research (e.g., Tomb et al., 2002), in showing that choices that result in larger rewards also result in larger SCRs.

To investigate whether rSCRs distinguished between selections prior to or following the display of knowledge a 4 × 2 (Deck by Time) repeated-measures design ANOVA was conducted. As no group differences were discovered in the initial analysis Group was removed as a factor in subsequent analyses. Missing values were imputed as in the aSCR analysis. The same results were found when participants with missing data were excluded.

An interaction between Deck and Time was found, *F*_(2.19, 56.97)^1^_ = 3.99, *MSE* < 0.01, *p* < 0.05. As with the overall analysis a main effect of Deck was found, *F*_(2.13, 55.46)_ = 3.77, *MSE* < 0.01, *p* < 0.05, but there was no effect of Time, *F*_(1, 26)_ < 1.0, *p* > 0.05. Figure 8B displays the mean rSCRs pre- and post-knowledge in each deck. The interaction between Deck and Time appears to be because rSCRs in the post-knowledge period for the advantageous decks are lower than the disadvantageous decks. In order to examine this further, the data were collapsed across Deck to provide values for the advantageous and disadvantageous decks in each time period and an interaction contrast was performed. This is effectively a 2 × 2 (Deck Type by Time) repeated-measures ANOVA, and revealed a significant interaction between Deck Type and Time, *F*_(1, 26)_ = 9.01, *MSE* < 0.01, *p* < 0.01; a main effect of Deck Type, *F*_(1, 26)_ = 11.96, *MSE* < 0.01, *p* < 0.01; but no effect of Time, *F*_(1, 26)_ < 1. Subsequent simple comparisons found a difference between Deck Types in the post-knowledge period, *F*_(1, 26)_ = 14.29, *MSE* < 0.01, *p* < 0.1, and not in the pre-knowledge period, *F*_(1, 26)_ < 1. In the selections after knowledge is displayed participants' physiological reactions following reward distinguish between the good and bad decks.

Figure 8C presents rSCRs for the participants who did not display knowledge. Here the pre- and post-knowledge periods are based on the mean values from the participants who did display knowledge. The early period includes the trials up to trial 39 and 43 for participants in the Specific and General groups, respectively. The late period includes all the subsequent trials. The mean values depicted in this Figure are much lower than those for participants with knowledge, suggesting that knowledge, and physiological activity may be linked. A similar pattern of reduced physiological activity in the post-knowledge period in decks C and D is also found in this group as in the participants with knowledge, but here it is also found for deck B. A 4 × 2 (Deck by Time) repeated-measures ANOVA was also conducted on this data. There was no interaction between Deck and Time, *F*_(3, 12)_ = 1.31, *MSE* < 0.01, *p* > 0.05; no main effect of Deck, *F*_(3, 12)_ = 1.54, *MSE* < 0.01, *p* > 0.05; and no main effect of Time, *F*_(1, 4)_ < 1. This result supports the conclusion from the analysis of the with-knowledge group that knowledge influences physiological activity. However, this conclusion is qualified by the low number of participants included in this analysis.

Figure 9A shows pSCRs over all selections and all participants. Mean pSCRs are higher in the decks with low frequency of punishment (B and D). Mean pSCRs are also higher than mean rSCRs. A 4 × 2 (Deck by Group) mixed-factor ANOVA revealed no interaction, *F*_(3, 90)_ < 1 and no main effect of group, *F*_(1, 30)_ < 1, thus replicating the other SCR data that found no group differences in SCRs. A main effect of Deck was found, *F*_(2.12, 63.66)1_ = 4.40, *MSE* < 0.01, *p* < 0.05. Subsequent simple comparisons revealed that pSCRs following selections from deck A were significantly lower than those from deck B, *F*_(1, 30)_ = 6.73, *MSE* < 0.01, *p* < 0.05; as were selections from deck C, *F*_(1, 30)_ = 10.02, *MSE* < 0.01, *p* < 0.05; while pSCRs for deck D were also significantly higher than those from deck C, *F*_(1, 30)_ = 5.73, *MSE* < 0.01, *p* < 0.05. There was no difference in pSCRs following selections from decks B and D, *F*_(1, 30)_ = 2.96, *MSE* < 0.01, *p* > 0.05, nor between decks A and D, *F*_(1, 30)_ = 2.96, *MSE* < 0.01, *p* = 0.10, which replicates Crone et al. (2004) and supports their conclusion that it is the magnitude of punishment and not the frequency that is influential for pSCRs.

Due to the infrequent nature of punishment relative to reward in all of the decks (far greater in decks B and D), many participants received no punishment in the post-knowledge period on some decks either as a result of not choosing them or because no punishment resulted from their choices. As this applied across so many participants a 4 × 2 (Deck by Time) analysis became impractical with the addition of missing values reaching unacceptable levels. However, the question of interest was whether physiological activity distinguished between the decks prior to a display of knowledge. As such pSCRs were averaged within participants in two ways. First, the mean pSCR for the advantageous and disadvantageous decks in the pre- and post-knowledge period were calculated for each participant. Figure 9B displays these means for those participants who displayed knowledge. A 2 × 2 (Deck Type by Time) repeated measures ANOVA, equivalent to that performed on the rSCR data, revealed a significant interaction between Deck Type and Time, *F*_(1, 26)_ = 4.44, *MSE* = 0.02, *p* < 0.05; but no main effect of Deck Type, *F*_(1, 26)_ < 1; nor a main effect of Time, *F*_(1, 26)_ = 1.96, *MSE* = 0.02, *p* > 0.05. Subsequent simple comparisons revealed that pSCRs were higher for the disadvantageous decks prior to knowledge being displayed than in the period afterward, *F*_(1, 26)_ = 6.04, *MSE* = 0.01, *p* < 0.05.

Second, the mean pSCRs for the decks with frequent and infrequent punishments were also calculated in each knowledge period. A 2 × 2 (Punishment Frequency × Time) repeated measures ANOVA found no interaction, *F*_(1, 26)_ < 1; no main effect of Punishment Frequency, *F*_(1, 26)_ < 1; and no main effect of Time, *F*_(1, 26)_ = 1.96, *MSE* = 0.02, *p* < 0.05. This result contrasts with Crone et al. (2004) who found higher pSCRs following choices from decks B and D.

Similar analyses were carried out for the participants who showed no knowledge. Figure 9C displays the mean values of pSCRs collapsed across the advantageous and disadvantageous decks up to and after the mean trial at which participants with knowledge displayed that knowledge. The 4 × 2 (Deck Type by Time) ANOVA revealed no interaction, *F*_(1, 26)_ = 1.42, *MSE* < 0.01, *p* > 0.05; no main effect of Deck Type, *F*_(1, 26)_ < 1; and no main effect of Time, *F*_(1, 26)_ = 1.11, *MSE* < 0.01, *p* > 0.05. The Punishment Frequency × Time ANOVA also revealed no interaction, *F*_(1, 26)_ = 1.43, *MSE* < 0.01, *p* > 0.05; no main effect of Deck Type, *F*_(1, 26)_ < 1; and no main effect of Time, *F*_(1, 26)_ < 1.

### Summary

Overall, we found that participants have knowledge about IGT contingencies sufficient to guide advantageous deck selection before the task's halfway point. We found no evidence of anticipatory autonomic activity that differentiated between deck types prior to this knowledge emerging. Differences in post-selection SCRs between deck types were found. Reward SCRs distinguished between the advantageous and disadvantageous decks across the whole experiment but only in participants who displayed knowledge and then only in later trials following their display of knowledge. Punishment SCRs were found to be larger for the disadvantageous decks in the pre-knowledge period but, again, only for participants who displayed knowledge.

## Discussion

We report an experiment in which we examined the claim that differential autonomic activity between deck types precedes the emergence of knowledge sufficient to guide behavior on the IGT. In contrast to previous research (Bechara et al., 1997) we found no evidence of differential pre-selection autonomic activity. These results replicate previous findings that differential aSCR activity is not necessary to succeed on the IGT (Gutbrod et al., 2006). In the absence of differential aSCR activity healthy participants learned to select advantageously on the IGT and developed knowledge of the task contingencies sufficient to guide behavior after approximately 40 trials. Our results suggest that aSCRs are not an unconscious measure of knowledge that predicts the choices people make.

Although we found that aSCRs do not differentiate between deck types prior to knowledge being displayed, a difference between deck types found over all rSCRs was localized within participants who displayed knowledge in the period following that knowledge being displayed. This result provides qualified support for the influence of knowledge rather than autonomic activity in influencing behavior on the IGT. The absence of any difference in aSCRs is problematic as a null effect can never be evidence for any hypothesis, and the results from the pSCRs suggest physiological responses occur for larger primary punishers but only in the initial period of the task. One possibility is that pSCRs did not distinguish between decks in the post-knowledge period because participants were aware that those decks had the worst losses. Alternatively the pre-knowledge pSCRs might influence subsequent decisions and constitute the first stage in a process toward somatic markers. This position is supported by the absence of these effects in participants who displayed no knowledge. So the physiological results are ambiguous showing that differences in post-selection SCRs emerge following knowledge for rewards but prior to knowledge for punishments. It could be argued that the post-knowledge difference in rSCRs indicates relief at escaping from a choice on a disadvantageous deck without a punishment. This would reflect the influence of knowledge. After all, these decks are more risky than the advantageous decks. Differential SCR activity, including aSCRs, may just reflect this awareness of risk.

Both Campbell et al. (2004) and Kleeberg et al. (2004) have reported failures to replicate the aSCR difference between deck types reported by Bechara et al. (1997). We also found that aSCRs did not increase over time replicating earlier results using a computerized version of the task (Suzuki et al., 2003; Carter and Smith Pasqualini, 2004). A possible explanation for the absence of differences in the aSCRs is the automated way in which they were gathered. The experimenter controlled the length of the inter-trial interval between SCR acquisitions in Bechara et al. (1997). This was to ensure that participants' physiological activity had returned to baseline following the previous choice. We did not employ exactly the same methods as Bechara et al. (1997) and so it is possible that as the inter-trial interval was fixed to a greater extent in the current experiment, physiological activity following the previous choice interfered with anticipatory physiological activity on the next choice. However, Crone et al. (2004) employed a similarly automatic methodology ensuring that the inter-trial interval was as long as reported by Bechara et al. (1997) and found similar results to theirs. The inter-trial interval in the experiment reported here was as long as the average reported by Bechara et al. (12 seconds). However, we found no differences in aSCRs following rewards or punishments. The results reported here show that the emergence of knowledge occurred at a similar point in the IGT as claimed by Bechara et al. (1997), but found no evidence for their claim that this was preceded by differential somatic activity. This has implications for Damasio's somatic marker hypothesis (SMH, Damasio, 1994, 1996). The SMH integrates emotional processing with rational decision-making positing a critical input from an embodied emotional system (somatic markers) in making decisions in complex and uncertain situations. As such, the IGT has been used extensively as a test of SMH. If accepted at face value our results are problematic for the SMH. Participants in this experiment improved on the IGT and displayed knowledge of which decks were worst in the long-run, yet the results suggest aSCRs played no part in this process. It may be that participants in this experiment did not have the same physiological reaction as those in other experiments but if this is the case it suggests that like other, clinical studies (North and O'Carroll, 2001; Heims et al., 2004) the absence of autonomic activity does not preclude learning on the IGT. Additionally, several studies (Hinson et al., 2003; Turnbull et al., 2003; Jameson et al., 2004) have shown that impairments in executive components of working memory detrimentally impact on IGT performance, suggesting that differences in aSCRs are driven by cognitive processes (implying knowledge) rather than vice versa. Alternatively, differential autonomic activity may have occurred in our sample, yet remained undetected because we used the relatively crude SCR measure. That we did not employ other measures of autonomic activity such as heart rate or respiratory response is a limitation of our study.

The results of this experiment are not only problematic for Bechara et al.'s (1997) account of IGT behavior. Knowledge sufficient to guide long-term advantageous selection emerged in the majority of participants at around the same time as Bechara et al. (1997) claimed. Participants were able to identify one of the best decks when initially questioned. As Maia and McClelland (2004) pointed out, unless losses have been experienced this will initially be deck A or B. But when losses begin to be encountered on these decks, they become disadvantageous, and it is then that participants have a problem keeping up. This was reflected in the assessment of participants' knowledge using either an aggressive or a conservative approach. For knowledge to be revealed using a conservative approach requires that knowledge to be present consistently across questioning and as losses are experienced on decks A and B, participants struggle to identify C and D as the new best decks. This time overlaps with when Bechara et al. (1997) claimed the aSCR difference emerged (trials 10–50). Kleeberg et al. (2004) reported that although they found no difference in aSCRs between deck types the increase in aSCR they observed averaged across all decks emerged between trials 20 and 40. These aSCR differences may be related to the shift in polarity of deck received values. The results from our study mean that Maia and McClelland's (2004) assertion that participants have knowledge sufficient to guide their behavior from the first questioning is supported, but unlike Maia and McClelland, our examination of participants' knowledge when their first losses on what become the disadvantageous decks are experienced, does not support the claim that this knowledge reflects the received deck contingencies. This also provides some support for the claim that failure to learn a successful strategy on the IGT may be linked to deficits in reversal learning (Rolls, 1999, 2005; Dunn et al., 2006).

As Maia and McClelland (2004) found, the assessments of participants' knowledge here sometimes indicated that their behavior did not reflect the knowledge that they possessed. Participants often did not select one of the best available choices despite the knowledge probes indicating that they were able to make this distinction. One explanation for this behavior is that their knowledge is not complete and few possess accurate knowledge of the deck contingencies. This makes non-optimal deck selection a reasonable option as participants attempt to explore the decks to learn more about their contingencies (Maia and McClelland, 2005). However, as Figures 4, 5 show, few participants come close to achieving this understanding. Indeed, most participants gave all the decks a negative rating suggesting that they were unaware that either decks C or D were profitable with repeated selection. This also suggests that for participants in this experiment the times when they lost money were most influential when they made their ratings. Certainly the pattern of changing selection from decks B and C driving learning observed in previous studies (Fernie and Tunney, 2006; Lin et al., 2007) was replicated here and was reflected in the question responses of participants given the Specific questions.

Persaud et al.'s (2007) claim that question style influenced awareness of deck contingencies is interesting in the context of our finding that participants' continued to select sub-optimally despite the presence of knowledge sufficient to guide behavior. There was no difference in when participants began to select advantageously between Persaud et al.'s groups demonstrating, surprisingly, that awareness, as measured with PDW, did not affect behavior. Regardless of whether PDW is an accurate measurement of awareness (Overgaard et al., 2010; Mealor and Dienes, 2012), Persaud et al.'s results seem to show that participants may have increased understanding of the task contingencies, or at least decreased uncertainty, following more specific questioning. However, Persaud et al. do not report on what degree of knowledge their participants possessed despite asking them the same questions we did. It may be that this increased knowledge, or decreased uncertainty, acted to reduce risk, or loss, aversion (Schurger and Sher, 2008) when wagering, but was not sufficient to reduce the exploratory behavior necessary to learn more about the task contingencies.

Our results suggest that participants do not generate anticipatory physiological activity sufficient to differentiate between deck types in the period prior to acquiring knowledge sufficient to guide their behavior. Knowledge required to profit on the IGT emerged later than claimed by Maia and McClelland (2004) but was not a complete understanding of the nature of the IGT. Indeed our results differed from those reported by both Maia and McClelland (2004) and Bechara et al. (1997). Both groups suggested that the majority of their participants end the experiment with conceptual knowledge of the IGT. We found little evidence in support of this conclusion, but conceptual knowledge was not critical for advantageous deck selection to occur.

### Conflict of interest statement

The authors declare that the research was conducted in the absence of any commercial or financial relationships that could be construed as a potential conflict of interest.
